# Solid-state NMR reveals differential carbohydrate utilization in diapausing *Culex pipiens*

**DOI:** 10.1038/srep37350

**Published:** 2016-11-17

**Authors:** James Chang, Jugeshwar Singh, Sungshil Kim, William C. Hockaday, Cheolho Sim, Sung Joon Kim

**Affiliations:** 1 Departments of Chemistry and Biochemistry, Baylor University, One Bear Place #97348, Waco, TX 76706, USA; 2 Biology, Baylor University, One Bear Place #97348, Waco, TX 76706, USA; 3 Geoscience, Baylor University, One Bear Place #97348, Waco, TX 76706, USA.

## Abstract

*Culex pipiens* is the mosquito that vectors West Nile Virus and other human-pathogenic flavivruses in North America. In response to shortened day length and lower temperatures, female *Cx. pipiens*e prepares for the diapause by actively feeding on carbohydrates to increase the biosynthesis of glycogen and lipid to store energy for overwintering. The effect of feeding different carbohydrates on glycogen and lipid biosynthesis in diapausing mosquitoes was investigated *in vivo* using 13C solid-state NMR. Diapause-destined adult females and nondiapausing counterparts after adult eclosion were fed with three different carbohydrate sources for 7 days: 1) 10% sucrose, 2) 10% D-[^13^C_6_]glucose, and 3) 1% D-[^13^C_6_]glucose co-provisioned with 10% sucrose. NMR measurements show that sucrose and glucose are metabolized differently in diapausing mosquitoes. Mosquitoes fed on sucrose primarily accumulate glycogen with increased branching structures, but less of lipids. In contrast, mosquitoes fed exclusively on glucose show accumulation of both glycogen and lipid with increased aliphatic chain length. Glucose is exclusively metabolized for the biosynthesis of triacylglyceride when mosquitoes were co-fed with sucrose. Our findings provide novel insights into the insect carbohydrate metabolism that governs glycogen and lipid biosynthesis during diapause, which is fundamental for the insect survival during inimical environments.

Diapause is a survival strategy that enables insects to cope with unfavorable environmental conditions or resource limitations^1^. Like many other temperate insect species, mosquito *Culex pipiens* enters an overwintering adult diapause in response to environmental cues such as shortened day length and decreasing temperatures. The timing of diapause is critical, as all the energy required for overwintering must be stored for the survival prior to entering diapause. Thus in preparation for winter, diapause-programmed females cease to seek blood meals and instead feed on nectar and other sugar sources^2^,^3^. This increased sugar uptake is converted into glycogen and lipid for energy storage^4^,^5^,^6^. Hypertrophy of adipocytes and elevation of stored fat start occurring within a week after adult eclosion in diapausing female, until the lipid reserves are two-fold higher than those found in their nondiapausing counterparts^7^. Figure 1 shows sucrose fed diapausing female with the enlarged abdomen with large fat storage cells. During winter, stored glycogen is utilized before lipids in fat body cells, and any remaining surplus lipids after overwintering are utilized for the production of eggs.

Since the uptake of carbohydrate is essential for the survival of diapausing mosquitos, different carbohydrate sources based on their habitats may contribute to variations in glycogen and lipid accumulation in diapausing mosquitoes^8^,^9^,^10^. The most common sugar source for mosquitoes is floral nectar, which consists primarily of three sugars: glucose, fructose, and sucrose^11^. Glucose and fructose are hexose monosaccharides, whereas sucrose is a non-reducible disaccharide of glucose and fructose (Fig. 1c). Relative amounts and concentrations of each carbohydrate can vary among species^12^. As these sugars are involved in signaling plant development^13^,^14^, the composition of carbohydrates in nectar can differ in response to the change in season and may pose a significant impact on diapausing mosquitoes. In response to temporal and dynamic changes in carbohydrate compositions of nectar, dipausing female mosquitoes are likely to have evolved to adopt a metabolic strategy that optimizes the utilization of most abundant carbohydrate for maximum nutrient (energy) storage, which increases the chance of survival for females through overwintering. Hence the underlying difference in metabolic requirements between males and females is reflected in their preference for different nectar sources they exhibit in the laboratory environment, where diapausing females tend to aggregate to flowers with nectar that are rich in monosaccharides (glucose and fructose)^15^ while males tend to favor flowers with high sucrose concentration nectar^15^,^16^. However, the effect of variation in the carbohydrate composition (glucose, fructose, and sucrose) of nectar on lipid and glycogen storage in diapausing mosquitoes has remained elusive^17^.

Here we investigate the effect of selective carbohydrate feeding by diapausing *Cx. pipiens* on glycogen and lipid accumulation. Diapause-destined adult females and their nondiapausing counterparts were ^13^C-isotope labeled (Fig. 1c) by feeding with three carbohydrate sources for 7 days after adult eclosion: 1) 10% (10 g/100 mL) sucrose (^13^C-natural abundance), 2) 10% D-[^13^C_6_]glucose, and 3) 1% D-[^13^C_6_]glucose co-provisioned with 10% sucrose. Accurate *in situ* quantifications of the glycogen and lipid accumulation in whole mosquitoes were performed by ^13^C solid-state NMR spectroscopy in order to determine effects of feeding different carbohydrates on glycogen and lipid accumulation. Our finding provides novel insights into the insect carbohydrate metabolism that governs glycogen and lipid biosynthesis during diapause, which is fundamental for the survival of insects in inimical conditions.

## Results and Discussion

### Adipocyte staining of diapausing *Cx. pipiens*

**Figure 1:**
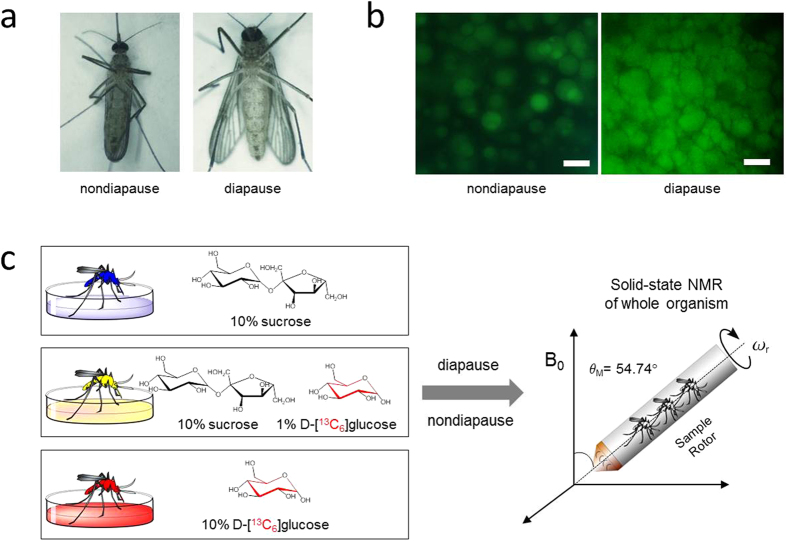
^13^C-Isotope labeling of *Culex pipiens* for solid-state NMR analysis. (**a**) Female *Cx. pipiens* fed with 10% (10 g/100 mL) sucrose post 7-days adult eclosion in non-diapausing (left) and diapausing (right) states. Diapausing female post 7-days adult eclosion with a visibly enlarged abdomen (right). (**b**) Fluorescent microscope images of fat body cells. Fat body cells of diapausing female fed on 10% sucrose shows fat hypertrophy. Lipids in adipocytes were stained using BIODIPY 493/503 (green). Scale bars represent 50 μm. (**c**) ^13^C-isotope labeling scheme for solid-state NMR. Female *Cx. pipiens* after adult eclosion were fed for 7 days with 10% (m/m, 10 g/100 mL) unlabeled sucrose (natural abundance), ^13^C-isotope labeled 10% D-[^13^C_6_]glucose, or 10% sucrose (natural abundance) spiked with 1% D-[^13^C_6_]glucose. Intact whole organisms were lyophilized and packed into 7-mm zirconia rotor and spun at 5 kHz magic-angle spinning for ^13^C solid-state NMR analysis.

The short-day-reared diapausing *Cx. pipiens* females exhibit the typical enlarged abdomen compared to the long-day-reared nondiapausing females (Fig. 1a). The microscope image of BODIPY 493/503 stained adipocyte from the abdomen of diapausing female fed on 10% sucrose (10 g/100 mL) shows an increase in the total amount of lipids and number of lipid droplets found in fat bodies (Fig. 1b)^7^.

### Sucrose metabolism in diapausing mosquitoes

**Figure 2:**
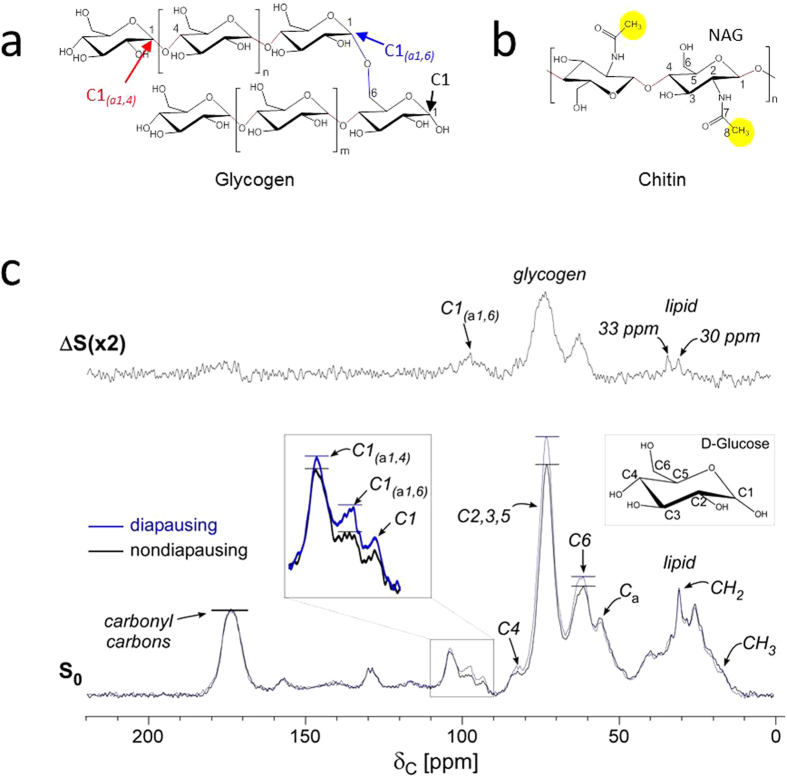
Natural abundance ^13^C-CPMAS spectra of female *Cx. pipiens*. (**a**) Chemical structure of glycogen. Linearly polymerized D-glucoses are connected by α (1 → 4) glycosidic linkages (red) with a branch connection by an α(1 → 6) glycosidic bond (blue). (**b**) Chemical structure of chitin. (**c**) Bottom: 75-MHz natural abundance ^13^C-CPMAS spectra of female *Cx. pipiens* in nondiapausing (black) and diapausing (blue) states after exclusively being fed 10% (10 g/100 mL) sucrose for 7-days post eclosion. All spectra are normalized to 175-ppm peak of peptidyl-carbonyl carbons for proteins. *O*-alkyl carbons of glycogen are visible at 60–105 ppm, lipid peaks at 20–40 ppm, and aromatics and unsaturated carbons at 130 ppm. Diapausing mosquitoes show increased *O*-alkyl carbons at 72 and 61 ppm. The figure inset shows enlarged 90–110 ppm range of the spectrum with increased anomeric carbons in diapausing mosquitoes consistent with glycogen accumulation. Diapausing mosquitoes show an increase in 98-ppm intensity corresponding to C1 carbon in α(1 → 6) glycosidic linkage. These changes indicate glycogen accumulation accompanied with an increase in branching structure. Top: Difference spectrum by the subtraction of ^13^C-CPMAS spectrum of nondiapusing from diapausing mosquitoes. Diapausing mosquitoes show only a small increase in lipid (CH_2_ at 30 ppm) in contrast with large glycogen accumulation (60–105 ppm).

Natural abundance ^13^C cross-polarization magic-angle spinning (CPMAS) spectra of nondiapausing (black) and diapausing (blue) female *Cx. pipiens* fed with 10% sucrose for 7-days post adult eclosion are superimposed and shown in Fig. 2c (bottom). Spectra are normalized to the 175-ppm intensity of peptidyl-carbonyl carbon of proteins and C7 of *N*-acetyl carbon in chitin (Fig. 2b)^18^. The general carbon chemical shift assignments are: i) carbonyl carbons at 170–180 ppm, ii) aromatic and ethylene carbons at 130 ppm, ii) anomeric carbons at 90–110 ppm, iii) *O*-alkyl carbons at 60–105 ppm, and iv) aliphatic carbons at 10–40 ppm. In diapausing mosquitoes, the *O*-alkyl carbon peak intensities at 61, 73, 82, 93, 99, and 104 ppm show a general increase. Chemical shift assignments for the *O*-alkyl carbons are shown in Fig. 2c where 61 ppm assigned to C6 of D-glucose (Fig. 2a) or *N*-acetylglucosamine (NAG) from chitin (Fig. 2b), and 73 ppm assigned to C2, C3, and C5 of D-glucose, or C3 and C5 of NAG. The C2 of NAG at 55 ppm is resolved from the C2 of D-glucose at 73 ppm, but overlaps with the Cα of proteins. The 82 ppm is assigned to C4 of both D-glucose and NAG^19^.

Chemical shift of the anomeric C1 carbon of D-glucose (Fig. 2c inset) is sensitive for different types of glycosidic linkages: i) from reducible sugars of monosaccharaides at 93 ppm, ii) branched D-glucose with α(1 → 6) linkage at 98 ppm, and iii) D-glucose connected by α(1 → 4) and β(1 → 4) at 104 ppm^20^. The increase in O-alkyl carbon intensities at 60–105 ppm in diapausing mosquitoes (Fig. 2c, blue) is due to the accumulation of glycogen and not from increase in chitin, as evidenced by absence of increase in acetyl-methyl carbon (C8) of NAG in chitin (Fig. 2b, yellow circles) resonating at 22 ppm^18^. As pupal cuticular proteins have been shown to increase in *Cx. pipiens* during the early stage of diapause^21^, cytoskeletonal reorganization may occur in preparation for overwintering. However, the net change in chitin biosynthesis in diapausing mosquitoes is small in comparison to the amount of large excess glycogen accumulation as observed by the increase in *O*-alkyl carbon intensities at 60–105 ppm. Figure 2c inset shows that diapausing mosquitoes have increased 99-ppm intensity corresponding to the branched C1 carbon with α(1 → 6) glycosidic linkage. Hence sucrose-fed diapausing mosquitoes store glycogen with increased branching structures. Glycogen accumulation is clearly visible in the difference spectrum (*ΔS*) obtained by subtracting the ^13^C-CPMAS spectrum of nondiapausing mosquitoes from diapausing ones (Fig. 2c, top). Compared to glycogen, aliphatic carbons at 30 and 33 ppm corresponding to CH_2_ in lipids show a smaller increase. These differential increases indicate that sucrose is preferentially routed to glycogen biosynthesis in diapausing mosquitoes.

### Glucose metabolism in diapausing mosquitoes

**Figure 3:**
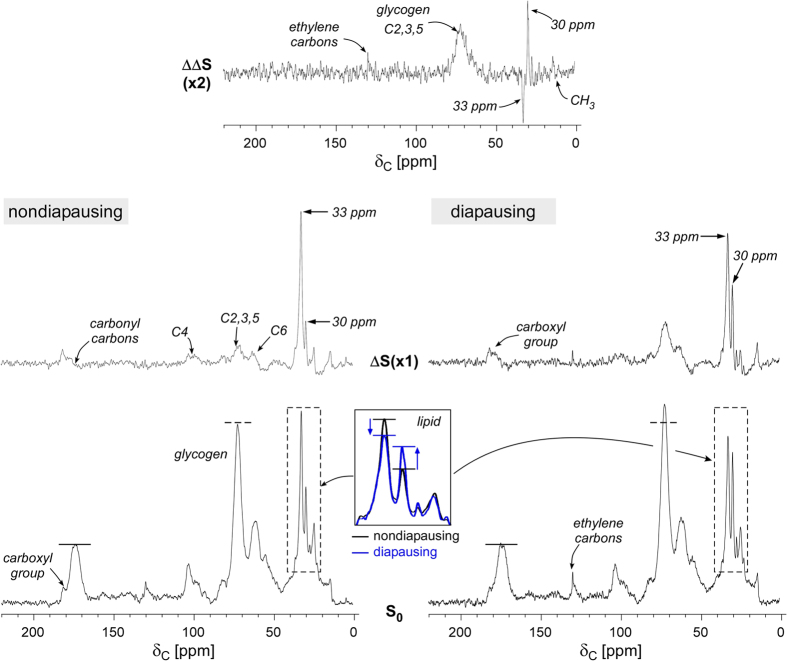
^13^C-CPMAS spectra of 10% D-[^13^C_6_]glucose fed female *Cx. pipiens*. Bottom) 75-MHz ^13^C-CPMAS spectra of nondiapausing (left) and diapausing (right) female *Cx. pipiens* fed with 10% D-[^13^C_6_]glucose as the sole-carbon source for 7-days post adult eclosion. The sample weights for D-[^13^C_6_]glucose-labeled nondiapausing mosquitos was 29.9 mg, and 39.4 mg for diapausing mosquitoes. The spectrum of nondiapausing mosquitos (left) was a result of 10000 acquisitions, and for diapausing mosquitos 10240 acquisitions (right). Middle) Difference spectra (*ΔS*) for nondiapausing (left) and diapausing (right) mosquitoes are obtained by subtracting the natural abundance CPMAS spectrum of nondiapausing mosquitoes from 10% D-[^13^C_6_]glucose-labeled CPMAS spectra. Top) Double-difference spectrum (*ΔΔS*) is obtained by subtracting the nondiapausing *ΔS* spectrum from the diapausing *ΔS* spectrum. Increased glycogen and lipid accumulations in diapausing mosquitoes are clearly visible in the *ΔΔS* spectrum.

^13^C-CPMAS spectra of nondiapausing (Fig. 3, lower left) and diapausing (Fig. 3, lower right) female *Cx. pipiens* fed exclusively on 10% D-[^13^C_6_]glucose for 7 days after adult eclosion demonstrate that glucose carbon is rapidly incorporated into both polysaccharides (60–110 ppm) and lipids (15–40 ppm). Unlike ^13^C-natural abundance spectra of sucrose-fed mosquitoes shown in Fig. 2c, ^13^C spectra of mosquitoes exclusively fed on 10% D-[^13^C_6_]glucose show increased CH_2_ intensities at 33 and 30 ppm (Fig. 3, dotted boxes) independently of diapause, indicating that glucose is preferentially metabolized for lipid biosynthesis in both diapausing and nondiapausing female *Cx. pipiens*. The relative peak-intensity ratio of glycogen at 76 ppm (dotted line) to lipid (30 ppm) in 10% D-[^13^C_6_]glucose-fed mosquitoes is approximately 1:1, whereas it is 2:1 in sucrose-fed mosquitoes shown in Fig. 2c. Therefore, mosquitoes exclusively fed on D-glucose show increased accumulations of both glycogen and lipid, while sucrose-fed mosquitoes show primarily glycogen accumulation.

Mosquitoes fed exclusively on 10% D-[^13^C_6_]glucose display a sharp ethylene carbon (-HC=CH-) resonance at 130 ppm. A sharp resonance of highly mobile olefin carbons is indicative of unsaturated lipids in the lipid bodies of adipocytes. A carboxyl-carbon resonance at 180 ppm, which appears as the shoulder to 175-ppm peak, is consistent with the carboxyl-carbon of fatty acid as D-[^13^C_6_]glucose is metabolized for lipid biosynthesis. In both diapausing and nondiapausing mosquitoes, D-glucose is metabolized for the biosynthesis of both lipid and glycogen.

Both nondiapausing and diapausing mosquitoes fed with 10% D-[^13^C_6_]glucose have a different aliphatic lipid composition than sucrose-fed mosquitoes (Fig. 3, bottom inset). The relative CH_2_ peak-intensity ratio of 33 to 30 ppm for nondiapausing mosquitoes (black) is 1.7 and for diapausing (blue) 1.1. In diapausing mosquitoes, 33-ppm peak intensity decreases concomitantly with 30 ppm peak intensity increase, whereas for mosquitoes exclusively fed on 10% sucrose, the 33 to 30 ppm ratio is constant at 0.6 for both diapausing and nondiapausing mosquitoes.

Difference spectra (*ΔS*) shown in Fig. 3 (middle) are the result of spectral subtraction of ^13^C-natural abundance CPMAS spectrum of 10% sucrose-fed nondiapausing mosquitoes (Fig. 2c, black) from the ^13^C spectrum of 10% D-[^13^C_6_]glucose-fed mosquitoes. Spectra are normalized to 175-ppm intensity prior to the subtraction. The spectral subtraction removes contributions from natural-abundance ^13^C, revealing the ^13^C distribution pattern solely from D-[^13^C_6_]glucose metabolism. Chitin biosynthesis is not involved in the metabolism of D-[^13^C_6_]glucose as evidenced by the absence of 55 ppm for C2 NAG in *ΔS* spectra. Differences between the *ΔS* of nondiapausing and diapausing mosquitoes are: i) more glycogen is accumulated in diapausing mosquitoes, ii) the olefin carbon of triacylglycerides (TAG) at 130 ppm is only visible in the *ΔS* of diapausing mosquitoes, and iii) diapausing mosquitoes show simultaneous increase in aliphatic CH_2_ carbon intensity at 30 ppm and decrease in 33-ppm intensity, which suggests an increase of lipid chain length in diapausing mosquitoes.

The difference in D-[^13^C_6_]glucose metabolism between diapausing and nondiapausing mosquitoes is further highlighted in the double-difference spectrum (*ΔΔS*) of Fig. 3 (top). The *ΔΔS* is obtained by subtracting the *ΔS* of nondiapausing from *ΔS* of diapausing mosquitoes. The *ΔΔS* spectrum shows that diapausing mosquitoes have increased intensities of *O*-alkyl carbon at 73 ppm, CH_3_ at 15 ppm, CH_2_ at 30 ppm, and olefin carbon at 130 ppm. The only peak decreases in intensity is the 33 ppm of CH_2_ positioned adjacent to either carboxyl carbons of lipid head group or olefin carbons in unsaturated lipids. Such changes indicate that D-[^13^ C_6_]glucose is metabolically routed preferentially to unsaturated aliphatic carbons during lipid biosynthesis. The absence of 180-ppm peak in *ΔΔS* suggests that the number of carboxyl-carbons in fatty acids are not increasing during diapause. This increase only in CH_2_ without a change in carboxyl-carbons of fatty acid is consistent with increased lipid chain length in diapausing mosquitoes.

### Glucose metabolism in the presence of sucrose in diapausing mosquitoes

**Figure 4:**
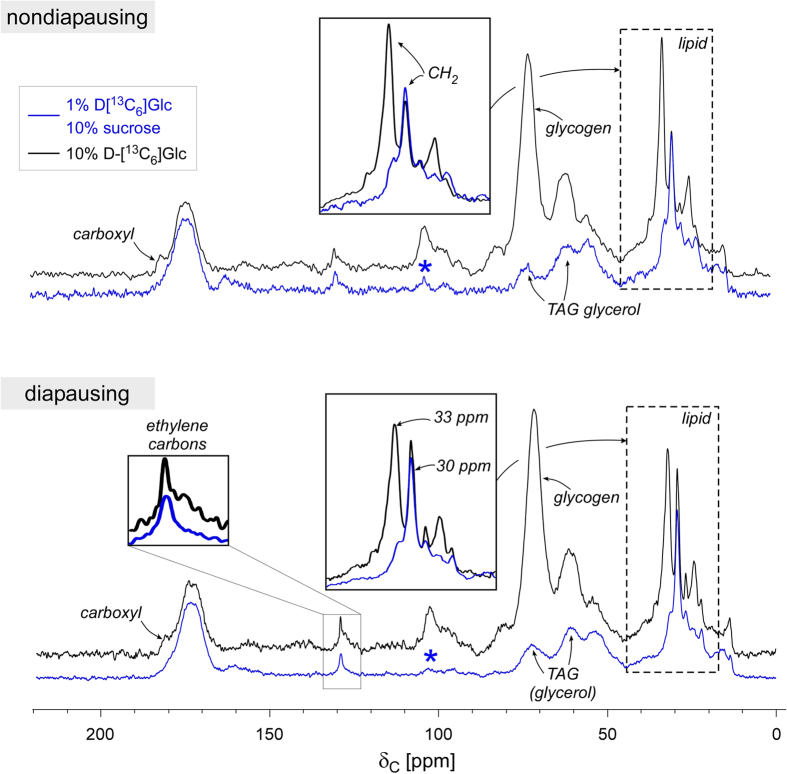
^13^C-CPMAS spectra of 1% D-[^13^C_6_]glucose and 10% sucrose fed female *Cx. pipiens*. 75-MHz 13C-CPMAS spectra of nondiapausing (top) and diapausing (bottom) female *Cx. pipiens* fed with the mixture of 10% sucrose with 1% ^13^C-isotope labeled D-[^13^C_6_]glucose (blue) for 7-days post adult eclosion. Spectra are superimposed with ^13^C-CPMAS spectra of mosquitoes fed 10% sucrose (black). All spectra are normalized to 175-ppm intensity. Spectra of mosquitoes fed with the mixture shows D-[^13^C_6_]glucose was preferentially metabolized for the biosynthesis of triacylglycerol without glycogen accumulation.

Figure 4 (right) shows 75-MHz ^13^C-CPMAS spectra of diapausing (bottom) and nondiapausing (top) female *Cx. pipiens* fed on a sugar mixture containing 1% uniformly 13C-isotope labeled D-[^13^C_6_]glucose and 10% unlabeled sucrose (blue) for 7 days post adult eclosion. These spectra are overlaid with spectra from mosquitoes exclusively fed on 10% D-[^13^C_6_]glucose (black) as comparison. All spectra are normalized to 175-ppm intensity. Although all spectra result from the metabolism of same isotope-labeled D-[^13^C_6_]glucose, ^13^C-CPMAS spectra of mosquitoes fed on 1% D-[^13^C_6_]glucose in presence of 10% sucrose (blue) show a distinct ^13^C-spectral profile different from ones fed exclusively on 10% D-[^13^C_6_]glucose (black). Changes in the metabolic profile of D-[^13^C_6_]glucose utilization are thus being observed through differences in ^13^C-CPMAS spectra between diapausing and nondiapausing mosquitoes.

The first major difference is found in the lipid profile. Enlarged lipid region (Fig. 4 insets) shows *Cx. pipiens* fed exclusively on D-[^13^C_6_]glucose (black) have CH_2_ resonances at 33, 30, and 25 ppm, whereas mosquitoes fed mixed carbohydrates (1% D-[^13^C_6_]glucose with 10% sucrose) show primarily 30 ppm (blue). Chemical shift assignments for saturated CH_2_ is 30 ppm, CH_2_ positioned next to the methyl-terminal end (ω-1) is 25 ppm, and CH_2_ adjacent to the ethylene carbon is 33 ppm. Therefore mosquitos that were fed exclusively 10% D-[^13^C_6_]glucose (without sucrose) demonstrate that D-[^13^C_6_]glucose is routed to biosynthesis of both saturated and unsaturated lipid. In contrast, mosquitos fed mixed carbohydrates preferentially routed 1% D-[^13^C_6_]glucose to saturated lipid biosynthesis.

The second major difference is that mosquitos fed mixed carbohydrates (1% D-[^13^C_6_]glucose with 10% sucrose) did not allocate D-[^13^C_6_]glucose to glycogen storage. Lack of glycogen biosynthesis is evidenced by absence of anomeric carbon intensities at 100 ppm (Fig. 4, star) despite the visible accumulation of *O*-alkyl carbons at 73 and 65 ppm. These *O*-alkyl carbons are not from glycogen as they have the intensity ratio of 1:2 consistent with the glycerol accumulation. On the other hand, mosquitoes exclusively fed on 10% D-[^13^C_6_]glucose have 73 to 65-ppm intensity ratio of 2:1 indicative of glycogen accumulation. Thus, mosquitos that were fed mixed carbohydrates show that D-[^13^C_6_]glucose is preferentially metabolized to i) mobile olefin carbons at 130 ppm, ii) saturated aliphatic carbons at 30 ppm, and iii) glycerol carbons at 73 and 65 ppm. These changes are in agreement with the exclusive routing of D-[^13^C_6_]glucose for the biosynthesis of TAG when co-provisioned with 10% sucrose in both diapausing and nondiapausing mosquitoes but not with glycogen biosynthesis. Thus female *Cx. pipiens*, when fed mixed sugars, preferentially utilizes sucrose for glycogen and glucose exclusively for the lipid biosynthesis.

### Total glycogen and lipid quantifications in diapausing mosquitoes fed exclusively sucrose or glucose

**Figure 5:**
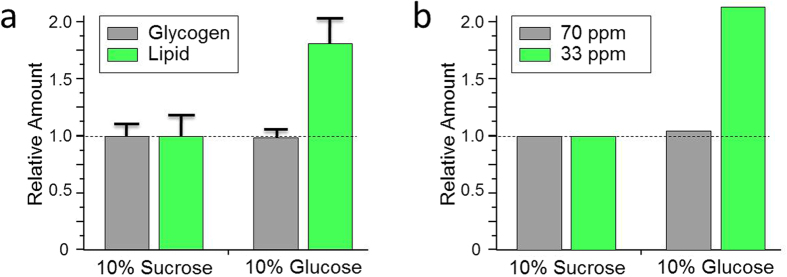
Lipid and glycogen quantifications of 10% sucrose and 10% glucose fed female *Cx. pipiens*. (**a**) Estimated total lipid and glycogen contents of individual diapausing females *Cx. pipiens* fed exclusively on 10% sucrose (left) and 10% glucose (right) for 7 days after adult eclosion as determined using methods described by Van Handel^22^,^23^. Total amounts of lipid (gray) and glycogen (green) per individual mosquitoes fed on 10% sucrose were normalized (dotted line) then compared with mosquitoes fed on 10% glucose. Error bars indicate standard error of mean. (**b**) Intensities of peaks at 70 and 33 ppm in ^13^C-CPMAS spectra of diapausing mosquitos fed exclusively on 10% sucrose (left) from Fig. 2, and on 10% D-[^13^C_6_]glucose (right) from Fig. 3. The 70-ppm intensity is proportional to the total carbohydrates (glycogen), and the 33-ppm intensity is proportional to the lipids in mosquitoes. The 70-ppm intensity (gray) and 33-ppm intensity (green) in mosquitoes fed with 10% sucrose were normalized. Feeding of different carbohydrate has a profound impact on the nutrient storage in diapausing mosquitoes. Mosquitoes fed exclusively on glucose show near doubling of lipid in comparison to mosquitoes fed solely on sucrose.

The total lipid and glycogen in individual diapausing females *Cx. pipiens* fed exclusively on 10% (10 g/100 mL) sucrose or 10% glucose for 7 days after adult eclosion were determined (Fig. 5a) using modified methods described by Van Handel^22^,^23^. The total lipid was estimated based on quantifying the reaction of phosphor-vanillin with unsaturated aliphatic chains primarily found in lipids by measuring the optical density (OD) at 525 nm. The total glycogen was estimated following acid hydrolysis of hexose by quantifying the hydroxy-methyl-furfural reaction through measuring the concentration of hydroxy-methyl-furfurylidene-anthrone with OD at 625 nm. Relative amounts of glycogen (gray) and lipid (green) in mosquitoes fed with 10% sucrose were normalized to compare against amounts found in diapausing mosquitoes exclusively fed 10% glucose. The amounts of glycogen in mosquitoes fed with sucrose or glucose were comparable, but the total amount of lipids nearly doubled in mosquitoes fed exclusively on glucose. Thus feeding of glucose increases the lipid biosynthesis in diapausing female *Cx. Pipiens*.

Total lipid and glycogen quantifications using methods described by Van Handel are in excellent agreement with the solid-state NMR result. Figure 5b shows the bar graph of 70 and 33 ppm intensities from ^13^C-CPMAS spectra of diapausing mosquitos that were exclusively fed 10% sucrose (left) and 10% D-[^13^C_6_]glucose (right). The 70-ppm intensity is directly proportional to the total carbohydrates (glycogen), and 33-ppm to lipids in the mosquito. The 70-ppm intensity (gray) and 33-ppm intensity (green) in mosquitoes fed with 10% sucrose were normalized. Mosquitoes that were fed exclusively on D-[^13^C_6_]glucose show the doubling of in lipids while the total glycogen remains unchanged.

### Differential carbohydrate utilization and overwinter survival

We found that glucose and sucrose are metabolized differently in diapausing mosquitoes. This observation is surprising because sucrose is not taken up directly to hemolymph in the form of a disaccharide following the ingestion, but hydrolyzed into fructose and glucose in the crop of foregut by α-glucosidases secreted from salivary glands^24^,^25^. Cleaved monosaccharides are then absorbed in the midgut and transported to the fat body for long-term storage, and the absorption of glucose at midgut does not distinguish glucose based on its origin. Hence, all glucose should share same metabolic fate whether it originated from sucrose or by direct ingestion. However, solid-state NMR results clearly show that glucose is metabolically discriminated based on its origin based on following observations: (i) diapausing mosquitoes exclusively fed on sucrose show excess accumulation in glycogen with increasing branching structure, but no significant lipid accumulation (Fig. 2), (ii) diapausing mosquitoes exclusively fed on glucose increase biosynthesis of both glycogen and lipids with longer fatty acid chain length (Fig. 3), and (iii) diapausing mosquitoes co-fed with 1% glucose in presence of 10% sucrose utilize glucose exclusively for TAG biosynthesis (Fig. 4).

Differential utilization of glucose and sucrose by *Cx. pipiens* provides an intriguing insight into fundamental mechanisms of carbohydrate metabolism in insects. Although the mechanism that governs differential carbohydrate utilization is unknown, we speculate that it is closely related with insect detecting the carbohydrate composition in the feed. Based on the composition, activation of insulin and adipokinetic hormones can elicit changes to metabolic homeostasis. For example, in *Drosophila* the gustatory receptor in brain can sense fructose in hemolymph and trigger the production of insulin-like peptides to regulate the glucose homeostatsis^26^. Similarly, sensing of glucose in the brain of *Drosophila* increases the activity of insulin producing cells^27^. Relationships between carbohydrate composition of the feed and induced insulin and adipokinetic hormone levels, as well as cascading events by the effector molecules on insect metabolic homeostasis, remain to be determined.

We propose that differential carbohydrate utilization in diapausing mosquito is the result of co-evolution with plants in response to seasonal changes in carbohydrate compositions of flower nectar. Flower nectar is one of the main carbon sources utilized by diapausing mosquitoes in preparation for winter. The nectar consists primarily of sucrose, fructose, and glucose; relative amounts of each carbohydrate can vary depending on the flower and seasonal conditions. Hence, female mosquitoes may have adopted a feeding strategy that efficiently utilizes abundant carbohydrates to maximize the long-term nutrient storage. Selectively feeding on nectar with the desired carbohydrate composition can have a profound impact on the long-term nutrient storage and survival. Consequences of this metabolism switching are reflected by diapausing female mosquitoes exhibiting preferential feeding of flower nectars that are rich in glucose and fructose^15^. In contrast, the males that do not undergo diapause preferentially feed on flower nectars rich in sucrose and thus do not compete against the females for same carbon source. This preferential feeding enables boost in lipid accumulation for diapausing females. Figures 4 and 5 show that female mosquitoes exclusively fed on glucose, as compared to mosquitoes fed exclusively on sucrose, gain approximately two-fold increase in lipid storage. The amounts of glycogen stored in both glucose and sucrose-fed mosquitoes were identical. Large excess lipid accumulation in glucose-fed mosquitoes likely increases their survival rate over sucrose-fed mosquitoes during prolonged overwinter. This advantage in survival is due to the stored glycogen being readily utilized during the first month of overwinter in diapausing mosquito^28^. Following the glycogen exhaustion, stored TAG in fat body is converted into free fatty acids and transported to mitochondria or peroxisomes for the ATP generation through beta-oxidation, and the lipid in fat body becomes the sole energy source for the remaining duration of diapause. Hence increased lipid accumulation in glucose-fed mosquitoes ensures greater chance of survival for diapausing mosquitoes during overwinter and surplus lipid for the egg production in spring.

## Materials and Methods

### Insect rearing and fat body staining

The stock colony of *Cx. pipiens* was reared at 25 °C and 75% relative humidity under a 15-h light:9-h dark (L:D) photoperiod as previously described^28^. When larvae reached the second instar, rearing containers were placed under one of two environmental conditions: nondiapausing females were generated by rearing at 18 °C, 75% relative humidity, and 15:9 L:D. To induce diapause, mosquitoes were reared at 18 °C, 75% relative humidity, and 9:15 L:D. To confirm diapause status, primary follicle and germarium lengths were measured, and the stage of ovarian development was determined according to the methods described by Christophers^29^.

Diapausing and nondiapausing adult females and their fat bodies were collected from 7-days post adult eclosion. Fat body cells were examined by staining fixed tissues with BODIPY 493/503 (Invitrogen). Stained fat body was examined with Zeiss Axioskop fluorescent microscope.

### ^13^C-isotope labeling of *Cx. pipiens*

Nondiapausing and diapausing female *Cx. pipiens* after adult eclosion were ^13^C-isotope labeled by feeding on sponges soaked with three different carbohydrates: 1) 10% sucrose (^13^C-natural abundance), 2) 10% (m/m, 10 g/100 mL) sucrose and 1% D-[^13^C_6_]glucose, 3) 10% D-[^13^C_6_]glucose for 7 days after adult eclosion. Isotope-labeled D-[^13^C_6_]glucose (uniformly ^13^C labeled with 99% enrichment) was purchased from Cambridge Isotope Laboratories, Inc. (Andover, MA). Following the 7-days post adult eclosion, mosquitoes were frozen at −80 °C then lyophilized. Lyophilized intact mosquitoes were weighed and packed into a 7-mm zirconia rotor for solid-state NMR analysis. The average dry weight of a mosquito was approximately 1.0 mg.

### Solid-state NMR spectrometer

Carbon-13 CPMAS NMR measurements were performed on 7.1-T (proton radio frequency of 300 MHz) Bruker Avance III with a double resonance HX probe. Lyophilized mosquitoes were contained in a 7-mm outer diameter zirconia rotor with Kel-F end-cap spinning at 5 kHz. Proton-carbon matched cross polarization was at 50 kHz with 2-ms contact time^30^. Spinning sidebands were suppressed using total-suppression of spinning sidebands sequence^31^ during echo in CPMAS. The proton dipolar decoupling was achieved by applying two-pulse phase modulation (TPPM15)^32^ on the ^1^H channel during acquisition. The π pulse length was 5 μs for ^13^C and the recycle delay was 3 s. The line broadening for spectrum was 20 Hz. ^13^C-PCMAS spectra were normalized to equal 175-ppm intensity of peptidyl-carbonyl carbons from proteins.

### Total lipid and glycogen quantification

Diapausing female *Cx. pipiens* after adult eclosion were fed two different carbohydrates for 7 days and frozen: 1) 10% (10 g/100 mL) sucrose or 2) 10% glucose. Total lipid and glycogen contents of individual mosquitoes were determined using modified methods described by Van Handel^22^,^23^. Briefly, total of 7–8 mosquitoes were measured per each treatment. For lipids, each sample had a single mosquito homogenized in 0.2 mL of chloroform:methanol (1:1) mixture. After evaporating the solvent, 0.2 mL of sulfuric acid was added, and samples were heated at 37 °C for 10 min. After cooling, 2 mL of vanillin reagent (0.12% vanillin in 68% phosphoric acid) was added. Samples were allowed to develop for 5 min, then the absorbance was measured at 525 nm. Sesame oil (Sigma, S3547) was used to generate a standard curve. For glycogen content, a single mosquito was homogenized in 0.2 mL of 2% sodium sulfate, followed by addition of methanol (1 mL) and centrifugation for 1 min. Supernatants were evaporated, and 2 mL of anthrone reagent (0.14% anthrone in 28% sulfuric acid) was added to each sample. Reactions were incubated at 37 °C for 15 min, then the absorbance was measured at 625 nm. Purified glycogen (Sigma, G0885) was used to generate a standard curve. Comparisons of different groups were carried out using the two-tailed unpaired Student’s t test. Differences were considered statistically significant at p < 0.05.

## Additional Information

**How to cite this article**: Chang, J. *et al.* Solid-state NMR reveals differential carbohydrate utilization in diapausing *Culex pipiens. Sci. Rep.* **6**, 37350; doi: 10.1038/srep37350 (2016).

**Publisher’s note:** Springer Nature remains neutral with regard to jurisdictional claims in published maps and institutional affiliations.
